# HOW TO MAXIMIZE THE REACH OF YOUR PUBLISHED WORK

**DOI:** 10.1093/geroni/igz038.823

**Published:** 2019-11-08

**Authors:** Sara McNamara

**Affiliations:** Oxford University Press, New York, New York, United States

## Abstract

Part 2 of this symposium will discuss how to maximize the reach and impact of your published work. Researchers are under increasing pressure to demonstrate the impact of their work. First, it is important to clearly articulate the translational impact of your work in the Discussion of your manuscript and briefly in the Abstract. In addition, it is important to know how to increase visibility of your manuscript because university administrators and funding agencies rely heavily on numerical metrics for decision-making. This presentation will look at the needs that metrics serve and provide an overview of how the dominant metric, the Impact Factor, is determined (along with a discussion of the metric’s flaws). The value of newer methodologies and alternative metrics (Altmetrics) will also be discussed. The presentation will also cover practical steps for how researchers can increase the reach and potential impact of their work.

